# Scalable and cost-effective Ag nanowires flexible transparent electrodes

**DOI:** 10.1039/c7ra13196h

**Published:** 2018-03-28

**Authors:** W. W. He, X. H. Yan, Y. M. Liang, Y. F. Long, C. Pan, J. L. Zhao, L. Chen, W. Xiong, Q. X. Liu

**Affiliations:** City College, Wuhan University of Science and Technology Wuhan Hubei 430083 China; State Key Laboratory of Materials Processing and Die & Mould Technology, Huazhong University of Science and Technology Wuhan 430074 China; Wuhan National Laboratory for Optoelectronics, Huazhong University of Science and Technology Wuhan 430074 China weixiong@hust.edu.cn; Urban Construction College, Wuhan University of Science and Technology Wuhan Hubei 430081 China liuqiuxin1956@sohu.com

## Abstract

Flexible transparent electrodes (TEs) are important for new electronic devices. This paper reports a scalable, cost effective Ag nanowires (AgNWs) TE, which is made of a SnO_2_·*x*H_2_O and AgNWs composite layer and a flexible polyethylene terephthalate (PET) bottom layer by a solution method at room temperature. The AgNWs/SnO_2_·*x*H_2_O composite TEs reveal a significant reduction of four orders in magnitude of sheet resistance, from 90 kΩ sq^−1^ to 12 Ω sq^−1^, while retaining transmittance of about 92% at 550 nm. This could be owing to the significant reduction of contact resistance for the weld-like junction of bound AgNWs. Compared with others, this method is characterized by filling gaps of the silver nanowire network with SnO_2_·*x*H_2_O. In addition, the adhesive forces between the AgNWs and the substrate are improved. This could be attributed to strong adhesion of SnO_2_·*x*H_2_O with the substrate. Moreover, this foldable transparent electrode is applicable for any non-planar surfaces and ultimately for future wearable optoelectronic devices.

## Abstract 2

This paper reports one of a scalable, cost effective Ag nanowires (AgNWs) TE, which reveals a significant reduction of four orders in magnitude of sheet resistance, from 90 kΩ sq^−^1 to 12 Ω sq^−^1, while keep transmittance of about 92% at 550 nm.

## Graphical abstract

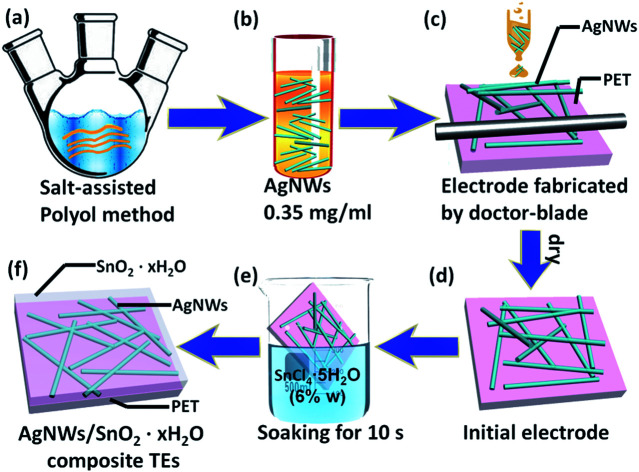

## 1. Introduction

A transparent electrode (TE) is an essential component for a wide variety of electronic devices, such as liquid crystal displays, touch screens, organic light emitting diodes (OLEDs), organic photovoltaics (OPVs), microchips, transparent heaters, and smart windows, *etc.*^1–7^ For electronic devices, indium tin oxide (ITO) with a market share of 93% is the most widely used TE.^8^ However, further application and development of ITO are expected to be receded due to three issues: scarcity of supply, expensive, and fragile ceramic nature. For these reasons, search for alternatives to ITO is motivated. Obviously, the ultimate market opportunity for alternative TEs is in fully flexible devices. To compete with ITO, the material for the alternatives should possess certain characteristics, for instance, outstanding performance (*e.g.* transmittance > 95% and sheet resistance < 100 Ω sq^−1^), flexible, good thermal-cycling, chemically stability, solution-phase coating processes, and cost-effective. Recently, carbon based TEs are the most promising alternatives, but still have problems of conductivity, mechanical properties and cost. In addition, metal TEs are also a candidate of the best alternatives. Metal nanowires electrodes can meet the requirements for the alternatives. The Ag nanowires (AgNWs) TE has performance even better than ITO,^9^ and is acknowledged as the proverbial “low-hanging fruit” for potential alternative to ITO.

However, pure AgNWs TEs also have three serious problems: low electrical conductivity, intrinsic nonconductive gaps which are not conductive and detrimental to the collection of carriers for the device, and poor adhesion to the substrate.

Firstly, pure AgNWs TEs still have electrical problems because of low contact between the AgNWs. As shown by experimental results, contact resistance of AgNWs (*R*_c_) is the main source of sheet resistance (*R*_sh_). The traditional methods for reducing *R*_c_ and improving conductivity are heating and pressing. Some of new methods are laser nano-welding, graphene coating, chemical annealing, plasmonic welding. However, all these methods only solve the problem of electrical conductivity but are helpless for addressing the other two issues.^10–17^

Secondly, pure AgNWs TEs have large area of blank space which is not conductive in AgNWs networks. In fact, AgNWs cover only 20% of the substrate. However, electrodes must cover all over the active areas for efficient charge extraction/injection because of the typically small lateral conductivity of organic semiconductor for some types of devices like OPVs and OLEDs. So, the concept of composite electrode is put forward. There are many methods to form a composite electrode with a silver nanowire, for example poly(3,4-ethylenedioxythiophene) polystyrene sulfonate (PEDOT:PSS) welding, graphene coating, double-layer structure, electrospun nanofibers, deposition of MnO_2_, TiO_2_, ZnO, AZO particles. The composite electrode can easily solve the problem of electrical conductivity and fill the gap between nanowires. Whereas PEDOT:PSS severely limits the device lifetime, and most of the nanoparticles need high temperatures (140 °C to 250 °C). Zilberberg *et al.* use sSnO_*x*_ sol–gel process to fabricate the TEs even at room temperature with the method of atomic layer deposition (ALD).^23^ But ALD limits the roll-to-roll production. So, currently, it still cannot achieve a large scale of cost-effective production.^10,15–23^

Thirdly, AgNWs TEs are easily to fall off the substrate due to the poor adhesion, which limits both of its usage and mass production. The composite electrode is very helpful to improve adhesion. However, the current technology is not suitable for roll-to-roll production.

In this work, we report a solution method to fabricate AgNWs TEs on PET substrate at room temperature which is suitable to the roll-to-roll production. More importantly, the fabrication processes are scalable and cost effective. With this method, the *R*_sh_ of the TEs can be reduced by four orders while maintaining high transmission. In addition, the gaps between the nanowires are successfully filled and the adhesion between the AgNWs and the substrate are improved. Moreover, this flexible transparent electrode is applicable for any flexible substrate and ultimately for future wearable optoelectronic devices. This will be of great significance to the extension and application of the electrode.

## 2. Experiment

### 2.1 Synthesis of AgNWs

**Fig. 1:**
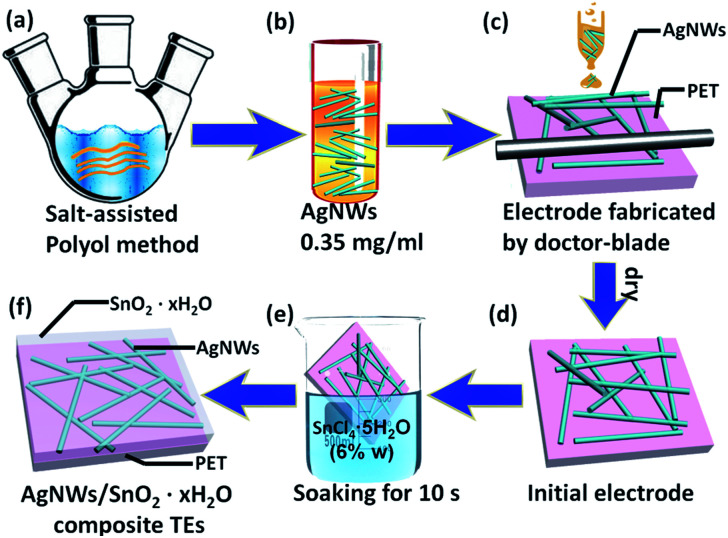
Flow chart of AgNWs/SnO_2_·*x*H_2_O composite TEs. (a) Prepare AgNWs by salt-assisted polyol method; (b) disperse AgNWs in ethanol of 0.35 mg mL^−1^ concentration; (c) Fabricate electrode by doctor-blade method; (d) initial electrode; (e) soaking treatment for 10 s; (f) schematic illustration of AgNWs/SnO_2_·*x*H_2_O composite TEs.

The AgNWs were synthesised by salt-assisted polyol method (Fig. 1a). 30 mL ethylene glycol (EG) solution of 0.052 mol L^−1^ AgNO_3_ and 0.067 mol L^−1^ PVP was thoroughly agitated. In addition, another 30 mL EG solution was prepared and heated at 130 °C for 1 h. 2.5 mL EG solution of 6 × 10^−4^ mol L^−1^ FeCl_3_ was dropped into the heated 30 mL EG. After 5–10 min, the mixed solution containing AgNO_3_ and PVP was added with a dropping speed of 0.5 mL min^−1^ into the heated solution. Finally, post-reaction solution was cooled naturally and then filtered by qualified filter paper (intermediate speed). AgNWs were washed out of the filter paper with deionized water. AgNWs were obtained by centrifugation at speed of 2000 rpm. Then the precipitation was retained, and the solution was replaced by ethanol. The step of centrifuge separation was repeated for three times.

### 2.2 Fabricate of AgNWs TEs

AgNWs were ultrasonically dispersed in ethanol with the concentration of 0.35 mg mL^−1^ (Fig. 1b). The AgNWs TEs were fabricated by the doctor-blade on the substrate of PET (Fig. 1c and d). And then the films were immersed in aqueous solution of SnCl_4_·5H_2_O (6% w) for 10 s. Finally, the TEs were washed with deionized water and dried.

## 3. Results and discussion

### 3.1 Conductivity

**Fig. 2:**
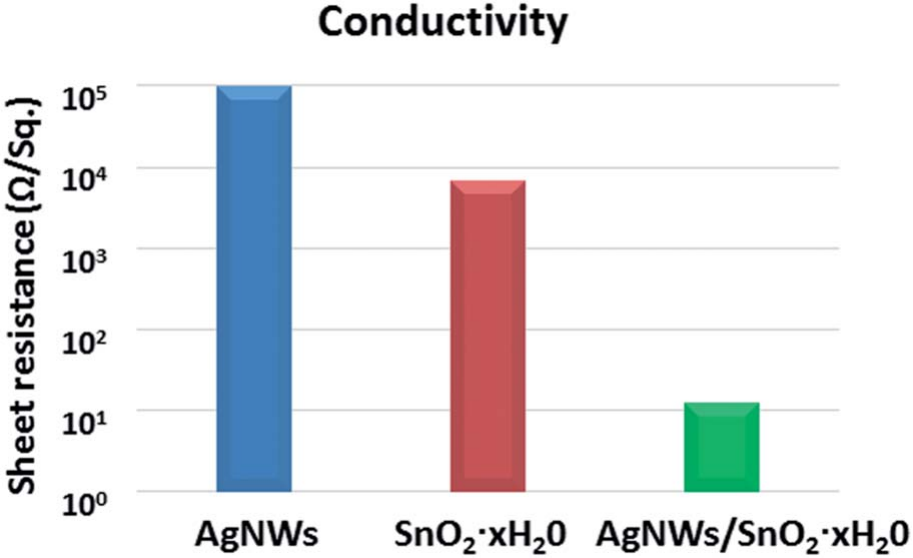
*R*_sh_ of initial AgNWs TEs, SnO_2_·*x*H_2_O film and AgNWs/SnO_2_·*x*H_2_O composite TEs.

The *R*_sh_ of the AgNWs TEs without any post-treatment are as high as 90 kΩ sq^−1^. This is a problem that AgNWs TEs usually have, mainly because AgNWs are piled up on the substrates without any contact. Therefore, the improvement of contact is very important for good conductivity. Fortunately, we found a scalable, and cost-effective way to solve this problem. The AgNWs TEs were immersed into the solution of SnCl_4_·5H_2_O (6% w) for 10 s. The *R*_sh_ of initial AgNWs TEs, SnO_2_·*x*H_2_O film and AgNWs/SnO_2_·*x*H_2_O composite TEs is shown in Fig. 2. The conductivity of the initial TEs is very poor with the *R*_sh_ over 90 kΩ (blue pillar). Similarly, the *R*_sh_ of the only SnO_2_·*x*H_2_O (red pillar) is 6 kΩ which is also too high for TEs. Fortunately, the *R*_sh_ of the AgNWs/SnO_2_·*x*H_2_O composite TEs (green pillar) is just 12 Ω sq^−1^. The *R*_sh_ of AgNWs TEs was reduced dramatically from 90 kΩ sq^−1^ to 12 Ω sq^−1^ by 99% or about 4 orders of magnitude.

To found the cause of the increase in conductivity, we analysed the solution. The solution of SnCl_4_·5H_2_O is not stable and easy to be hydrolysed. The reactions in the aqueous solution of SnCl_4_·5H_2_O can be expressed as:

1 SnCl_4_ + 6H_2_O → H_2_[Sn(OH)_6_] + 4HCl

2 SnO_2_·6H_2_O + 6HCl → H_2_SnCl_6_ + 8H_2_O

where H_2_[Sn(OH)_6_] in eqn (1) can be written as SnO_2_·6H_2_O which will react to HCl to obtain product H_2_SnCl_6_ that causes the solution to be acidic with pH value of 2, as confirmed through experiments. And because the number of Cl^−^ is smaller than Sn^4+^, there is still some residual of SnO_2_·*x*H_2_O. As a result, the hydrolysis of the solution finally obtained the SnO_2_ colloid and H_2_SnCl_6_.

There are two main effects of SnCl_4_·H_2_O solution on TEs, which lead to the *R*_sh_ reduction of four orders in magnitude, from 90 kΩ sq^−1^ to 12 Ω sq^−1^.

Firstly, SnO_2_·*x*H_2_O can make the nanowire in close contact and fill the gap of AgNWs TEs. The nanowires will be adsorbed on the substrate due to the strong adhesion of SnO_2_ with substrate. And adhesion of SnO_2_ with glass can even reach 20 MPa. SnO_2_·*x*H_2_O can help the nanowires adhere firmly to the surface of the substrate and contact closely to each other for better transmission of electrons. Moreover, the filling of the voids by SnO_2_·*x*H_2_O is very important for electronic devices. The filling of the nonconductive voids can improve the collection of carriers which will improve the performance of electronic devices.

Secondly, Cl^−^ ions could help to bind the AgNWs together and form a weld-like junction which will further increase the conductivity of the film. As discussed in previous studies,^21^ silver atoms in AgNWs may be dissolved slowly in the presence of Cl^−^ and dissolved oxygen in water. Due to redox reactions, it will produce solvated silver ions (Ag^+^). The redox reaction can be written as:

3 4Ag + O_2_ +2H_2_O ↔ 4Ag^+^ + 4OH^−^

We need to pay attention to the atomic oxygen on the silver surface. It may block the re-deposition of silver ions onto the silver surface. And atomic oxygen on the silver surface comes from molecular oxygen which is adsorbed and dissociated to atomic oxygen between 200 K and 500 K.^24^ However, concentration of dissolved oxygen in water is very limited so that Ag^+^ ions could redeposit onto the relatively more active AgNWs surface and junction. The deposition of Ag at the junction will allow the two nanowires to be successfully welded together and will reduce *R*_c_, which is the main source of *R*_sh_. So, the Cl^−^ in the solution can help improve the conductivity of TEs.

In Table 1, the electrical properties of different composite TEs are compared and analysed. The last row presents our results, whose perform is seen to be better than other types of electrodes. When the length of the nanowire is doubled, the resistance is reduced by an order of magnitude. In contrast, while the diameter varies at 50–100 nm, the transmittance is maintained at 90%, the longer the nanowires are, the better the electrical properties. And we also found that when the length is the same, the thinner the nanowire is, the better the electrical properties are.

**Table 1:** Comparison of electrical properties of composite TEs^25–29^

Composite TEs	Length (μm)	Diameter (nm)	*R*_sh_ (Ω sq^−1^)	Transmittance
AgNWs/PEDOT:PSS	20–30	50–100	600	90%
AgNWs/PEDOT:PSS	50–100	50–100	54	90%
AgNWs/RGO	10–20	50–100	20	92%
AgNWs/Aa-PDA	10–20	40–50	20	94%
AgNWs/sSnO_*x*_	10–20	50–100	5.2	87%
AgNWs/ZnO	5–10	50–100	4.2	85%
AgNWs/SnO_2_·H_2_O	10–20	50–100	12	92%

### 3.2 Optical properties

**Fig. 3:**
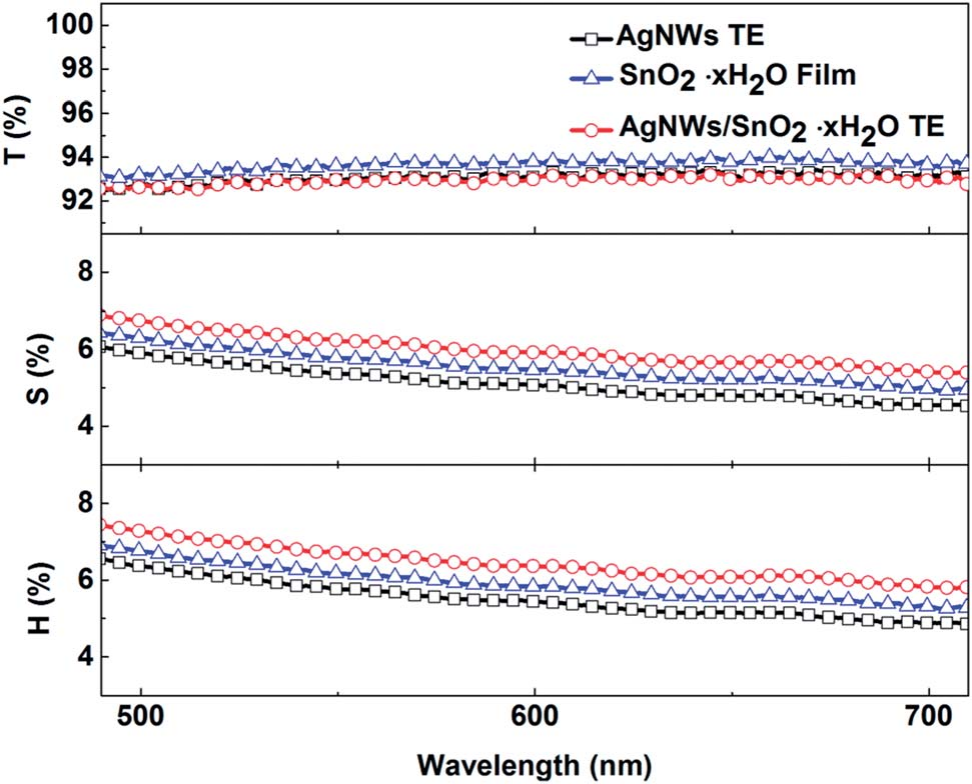
Transmittance (*T*), scattering (*S*), and haze (*H*) properties of initial AgNWs TEs, SnO_2_·*x*H_2_O film and AgNWs/SnO_2_·*x*H_2_O composite TEs.

Optical properties are also an important aspect of transparent electrodes. Fig. 3 shows the change of transmittance (*T*), scattering (*S*), and haze (*H*) properties of initial AgNWs TEs and AgNWs/SnO_2_·*x*H_2_O composite TEs. Haze is a percentage of the transmitted light intensity which is more than 2.5 degrees deviation from the incident light. And haze is equal to the percentage ratio of the scattered light flux (*S*) within large angles deviating from the incident direction by more than 2.5° to the transmitted luminous flux (*T*), *i.e.*
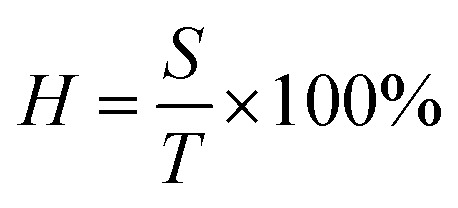
where *H* represents haze, and *S* represents the large-angle scattered light flux, and *T* represents transmitted luminous flux. The demand for haze is not the same for different applications. For display devices, haze should be as small as possible (usually <1%). Yet, for photovoltaic devices, haze can be as large as possible to increase optical path length in devices.

As shown in Fig. 3, for the initial AgNW TEs (black line), fabricated by means of the doctor-blade coating method, the transmittance is over 92.9% (at 550 nm wavelength). But the *R*_sh_ is too high for the pure AgNW TEs. Similarly, the only SnO_2_·*x*H_2_O film (blue line) has a transmittance of 93% but its conductivity is not good enough yet. In contrast, for the composite TE, its transmittance is little changed and remained at 92.7%, but its conductivity is significantly improved, also its haze and scattering are increased by about 1%. This means that the presence of the SnO_2_·*x*H_2_O layer has little effect on the transmittance but will enhance the scattering due perhaps to the change of refractive index.

### 3.3 Morphology

**Fig. 4:**
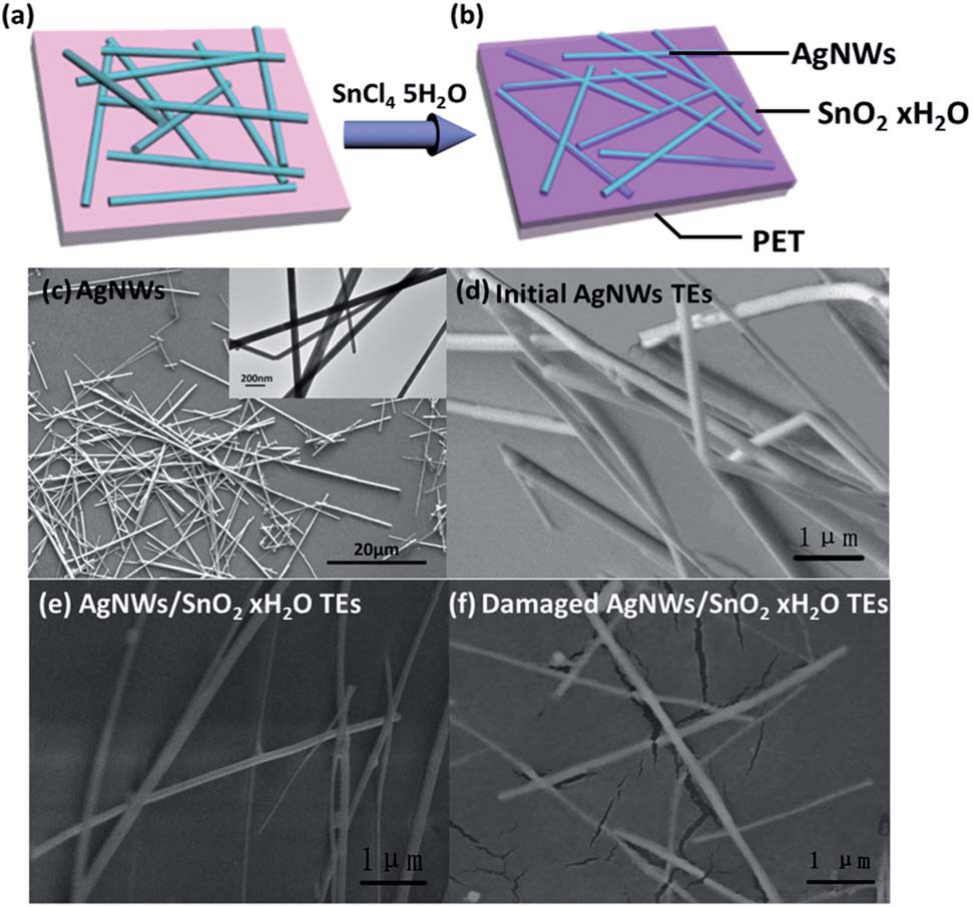
Diagram of electrode structure and SEM images. (a) Structure of the initial AgNWs TEs; (b) structure of the AgNWs/SnO_2_·*x*H_2_O composite TEs; (c) AgNWs and the TEM image of AgNWs (top right graph and inset picture); (d) the initial AgNWs TEs as looked from the side; (e) the AgNWs/SnO_2_·*x*H_2_O composite TEs, (f) the damaged AgNWs/SnO_2_·*x*H_2_O composite TEs (the composite TE is made on a flexible substrate and then elongated to lead to the tearing of the film).

Fig. 4a and b are the diagram of structure of the initial AgNWs TEs. Fig. 4c and d are the SEM image corresponding to the schematic in Fig. 4a. From Fig. 4c, we can see that the length of AgNWs is about 10–20 μm, and the diameter is 50–100 nm. As shown in Fig. 4d, AgNWs do not lie flat on the substrate, but tilt arbitrarily. The contact between AgNWs is poor, and some of the nanowires are even turned up. Accordingly, the large resistance of the initial AgNWs TEs probably comes from the poor contact. Therefore, to reduce *R*_sh_, we need to first reduce the *R*_c_. Fig. 4b and e are the schematic and SEM image of the composite TEs, respectively. Fig. 4e shows that SnO_2_·*x*H_2_O filled the gaps between nanowires or even covered the surface of the nanowires. Fig. 4f is a SEM image of damaged composite TEs which was made on a flexible substrate and pulled to split. As clearly seen through the tearing film in Fig. 4f, the gaps in the nanowire are really filled by the material. Fig. 4e and f show that nanowires are all compacted on the substrate and some nanowires are in good contact in AgNWs/SnO_2_·*x*H_2_O composite TEs.

**Fig. 5:**
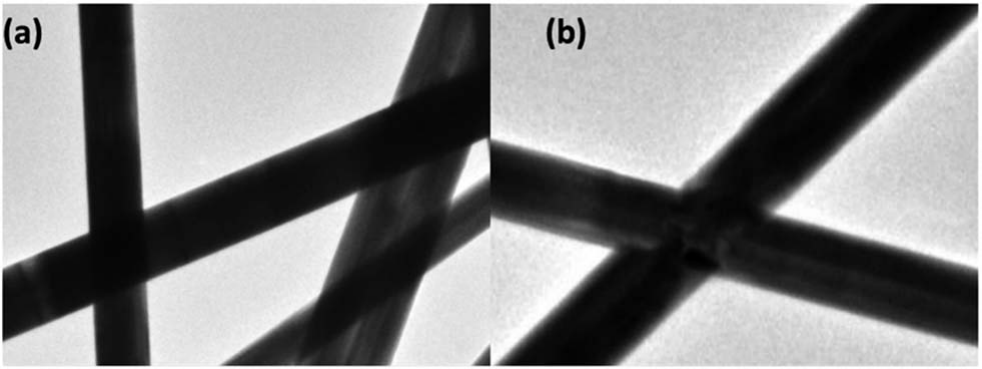
(a) TEM images of initial AgNWs crossing, (b) TEM images of weld-like AgNWs junction of AgNWs/SnO_2_·*x*H_2_O composite TEs.

The junction of the composite TEs is tested by TEM to confirm the contact of the nanowires. Fig. 5a and b show the TEM images of the crossing structure of initial TEs and the composite TEs. For initial AgNWs TEs (Fig. 5a), the connection between the crossed AgNWs is not welded. And the force between the nanowires is mainly van der Waals force. For the AgNWs/SnO_2_·*x*H_2_O composite TEs, the connection between the crossed AgNWs is welded. The force of the weld-like junction is intermolecular force. Therefore, the nanowires are firmly and stably welded together although exfoliated by the ultrasonic from the substrate. This means that the solution of SnO_2_·*x*H_2_O can help to improve contact of nanowires.

### 3.4 Chemical analysis

**Fig. 6:**
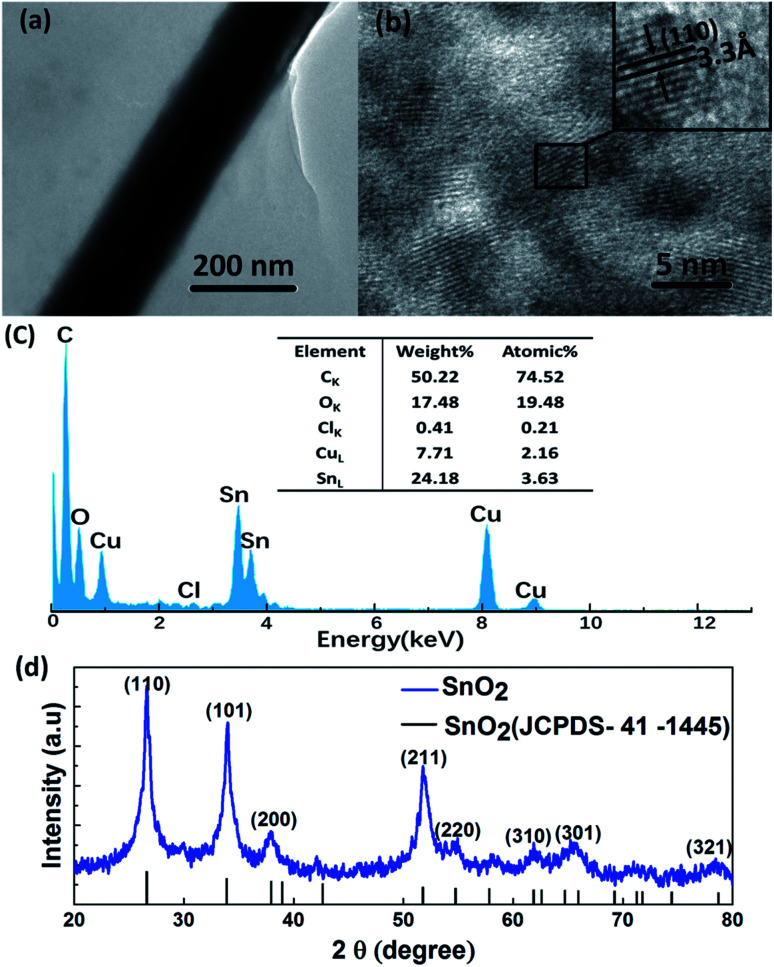
Microstructure of composite films. (a) TEM images of composite TEs, and (b) HRTEM image of SnO_2_ annealed at 400 °C for 30 min. (c) EDS spectrum of SnO_2_·*x*H_2_O along with the table listing the elements and their weight percentage and atomic percentage, (d) XRD patterns of crystallized SnO_2_ annealed at 400 °C for 30 min.

The chemical in the nanowire gap is characterized. Fig. 6a shows the TEM-image of the film. Obviously, there is a layer of material, which might be amorphous, deposited onto the surface of the TE and AgNWs. Based on the analysis of the components of the hydrolysis solution, the amorphous substance on the film surface are believed to be SnO_2_·*x*H_2_O.

The elemental composition is determined by Energy Dispersive Spectrometer (EDS). Fig. 6c shows the EDS spectrum of the amorphous, and reveals the presence of tin (Sn), oxygen (O), carbon (C), copper (Cu) and chlorine (Cl) elements in the sample. Carbon is the most abundant. This could be attributed to the amorphous carbon-coated copper grids, which is also the source of copper. The small amount of chlorine comes from the H_2_SnCl_6_ in the solution. The most important thing is verifying the existence of tin and oxygen in the amorphous thought to be SnO_2_·*x*H_2_O. To further confirm our speculation, we need to remove the crystalline water from the amorphous to verify the existence of SnO_2_. When SnO_2_·*x*H_2_O is annealed at 400 °C for 30 min, SnO_2_·*x*H_2_O will be turned into the crystallized SnO_2_. The crystallized SnO_2_ is characterized by high resolution transmission electron microscopy (HRTEM). The HRTEM image shows a clear lattice spacing of 3.3 Å in Fig. 6b, which exactly matches to the (110) plane of single crystal SnO_2_.

Fig. 6d presents the XRD patterns of this crystalline substance. The major XRD peaks at 2*θ* = 26.61, 33.89, 37.95, 51.78 and 65.94° are assigned to the (110), (101), (200), (211) and (301) reflection planes of tetragonal SnO_2_, respectively. The diffraction peaks of the products can be well indexed to the known tetragonal rutile SnO_2_ (JCPDS 41-1445), which confirms the presence of SnO_2_ in the annealed products. So far, the amorphous material is determined to be SnO_2_·*x*H_2_O.

### 3.5 Thermal stability

**Fig. 7:**
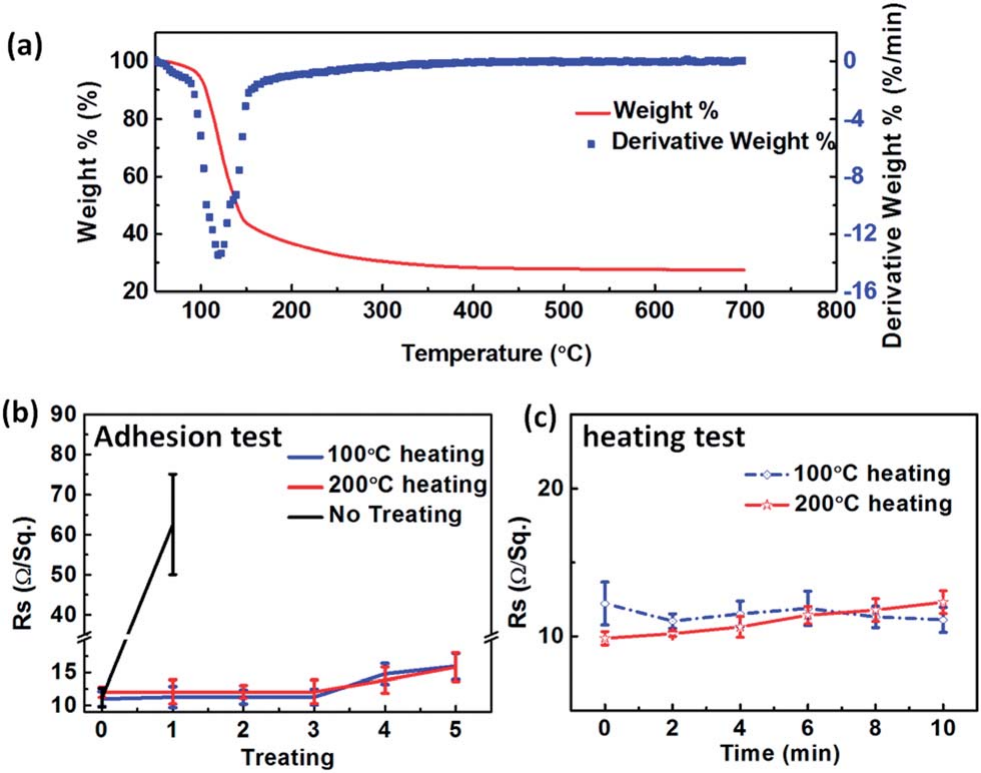
(a) Thermogravimetric analysis of SnO_2_·*x*H_2_O. (b) adhesion test of electrodes. The horizontal axis represents the number of adhesive tape. (c) Resistance change of AgNWs/SnO_2_·*x*H_2_O composite electrodes during heating.

The thermogravimetric analysis of SnO_2_·*x*H_2_O shows that SnO_2_·*x*H_2_O began to severe loss of water from 100 °C (Fig. 7a). Nevertheless, crystallization temperature of SnO_2_ is about 400 °C. If SnO_2_·*x*H_2_O is heated at 100 °C or 200 °C, we will get the amorphous substance which removed some of the crystal water.

This simple heat treatment surprisingly gives us an unexpected benefit that the adhesion of the film is significantly improved. As we all know, the thin film of AgNWs can easily be erased when we touch it gently due to the poor adhesion of the initial AgNWs TEs. However, there's always a way out! The adhesion of the composite electrodes is improved significantly just with a 100 °C or 200 °C heating treatment. This is attributed to the strong adhesive strength between SnO_2_ and the substrate. When the non-heated composite electrodes were taped and torn down just for one time, the *R*_sh_ increased from 12 to 60 Ω sq^−1^ which is increased by 5 times (black line in Fig. 7b). Nevertheless, the 100 °C or 200 °C heating treatment has only a little influence on the conductivity of the composite TEs (Fig. 7b). The *R*_sh_ increases only by 3 Ω sq^−1^. In addition, the thermal stability of the heated composite electrode is quite good. The *R*_sh_ of the film changes little but maintains at about 12 Ω sq^−1^ during heating (Fig. 7c). In conclusion, the adhesion of the composite TEs can be improved by heat treatment, and the TEs can also tolerate adhesive tape test.

**Fig. 8:**
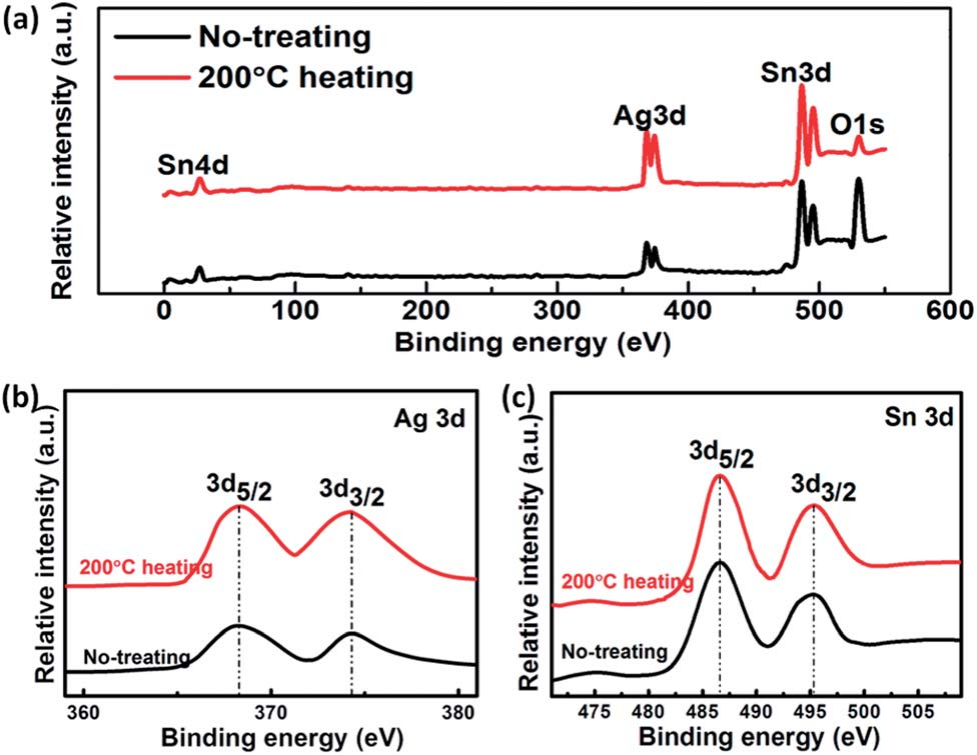
XPS spectra of SnO_2_·*x*H_2_O. (a) General XPS spectra of initial and 200 °C heating SnO_2_·*x*H_2_O. (b) Ag 3d XPS spectrum of SnO_2_·*x*H_2_O. (c) Sn 3d XPS spectrum of SnO_2_·*x*H_2_O.

XPS spectra are used to characterize the initial composite TEs and the heated composite TEs at 200 °C. We want to know whether the heat treatment has other effects on the electrode, in addition to the loss of crystalline water. Fig. 8 shows the X-ray photoelectron spectroscopy (XPS) of the composite TEs. The XPS survey spectrum (Fig. 8a) shows the peaks of Ag 3d doublet, Sn 3d doublet, Sn 4d and O 1s peaks. There are two peaks with binding energies of 368.3 eV for Ag 3d_5/2_ and 374.3 eV for Ag 3d_3/2_ in the regional spectrum (Fig. 8b) of Ag 3d. This is consistent with the standard binding energy of pure silver. The peaks at a binding energy of 486.6, 495.3 and 27.1 eV (Fig. 8c) can agree with the Sn 3d_5/2_, Sn 3d_3/2_ and Sn 4d_3/2_, respectively. Their standard combination can be very close to the previously reported results, indicating that the valence states of Ag and Sn are 0 and +4, respectively. This means that the heat treatment at 200 °C does not cause a change in the valence of the composite electrode.

## 4. Conclusions

As reported previously, AgNWs transparent electrode usually has the problems of poor initial conductivity and intrinsic gap between nanowires. This paper demonstrated a simple solution-processed method for a scalable, cost effective and flexible AgNWs TEs. Compared with others, this method is characterized by the gap of the silver nanowire network filled by SnO_2_·*x*H_2_O. The electrode forms a new kind of AgNWs/SnO_2_·*x*H_2_O composite structure just with 10 s immersion in solution of SnCl_4_·5H_2_O at room temperature. It is confirmed that aqueous solution of SnCl_4_·5H_2_O could help to reduce the *R*_sh_ by four orders of magnitude, from 90 kΩ sq^−1^ to 12 Ω sq^−1^, while keep transmittance of about 92% at 550 nm. In addition, the adhesive forces between the AgNWs and the substrate is improved. The optimization of the performance is attributed to the effects of SnCl_4_·5H_2_O solution which mainly contains Cl^−^ and SnO_2_·*x*H_2_O because of hydrolysis. There are two main effects on AgNWs TEs. Firstly, Cl^−^ can help to fuse the contact of nanowires which reduces *R*_c_ of TEs. Secondly, SnO_2_·*x*H_2_O can help to fill the space of networks. The conductivity of the composite TEs is improved attributed to this important filling. At the same time, the coverage on the active areas can improve the efficiency of charge extraction/injection for devices. Surprisingly, the adhesion of AgNWs TEs can be significantly improved with 100 or 200 °C heating. What's more, the simple solution-method at room temperature makes it possible for the roll-to-roll production.

## Conflicts of interest

There are no conflicts to declare.

## Supplementary Material
